# Developing a dashboard for use in a forensic and intensive care psychiatric unit: a quality improvement project

**DOI:** 10.1192/bjo.2021.528

**Published:** 2021-06-18

**Authors:** Keara Jamieson, Daniel Bennett

**Affiliations:** 1 University of Aberdeen; 2 University of Aberdeen, Royal Cornhill Hospital, NHS Grampian

## Abstract

**Aims:**

Dashboards provide a visual summary of relevant data to track performance against key indicators over time. They are used in healthcare to monitor the quality of patient care and to identify potential quality improvement projects. There is little published evidence of them being used in mental health services, especially in forensic psychiatric care.

This project aims to design a dashboard for use in a forensic and intensive psychiatric care unit, by specifying measures and ideal features it would include.

To develop a model for a quality dashboard for use

To decide which measures would be reported on the dashboard

To find reliable methods of assessing said measures

To explore staff preferences as to how the dashboard would display data, and how they would like the information to be disseminated

To use blank data to design a mock dashboard interface for feedback

**Method:**

A literature search was conducted on healthcare dashboards and quality improvement projects taking place on low-secure psychiatric wards similar to the Blair unit. Potential outcome measures and methods of assessing them were researched. Staff thoughts on the dashboard, and which measures they would like to see included, were explored in interviews and using a survey

**Result:**

Blank data were fed into excel to create example graphs for a mock dashboard. The results section details: measures to be included, such as staff turnover rate, absences, and patient satisfaction levels; how they can be assessed; and specific features of the dashboard, such as the capability to track trends in selected quality indicators over a period of time. Further development of this project out with the 4 week development timeframe will require cooperation from IT services and unit management staff.

**Conclusion:**

Many staff suggestions, whilst valuable measures, were more suitable for use in a clinical or nursing dashboard, rather than a quality dashboard. COVID-19 factored into reasons why staff requested certain measures, and also meant that less staff were available to be contacted about the project. This project has limitations based on the four-week timeframe, but could be further developed by staff on the unit if desired.

